# First-line Double Stentriever Thrombectomy for M1/TICA Occlusions: Initial Experiences

**DOI:** 10.1007/s00062-022-01161-2

**Published:** 2022-04-13

**Authors:** Pedro Vega, Eduardo Murias, Jose Maria Jimenez, Juan Chaviano, Jose Rodriguez, Sergio Calleja, Montserrat Delgado, Lorena Benavente, Maria Castañon, Josep Puig, Helena Cigarran, Faustino Arias, Rene Chapot

**Affiliations:** 1 Department of Radiology, Hospital Universitario Central de Asturias, Avda. Roma S/N 33011, Oviedo, Asturias, Spain; 2 Department of Neurology, Hospital Universitario Central de Asturias, Asturias, Spain; 3 IDI-Radiology, Doctor Josep Trueta University Hospital of Girona, Girona, Spain; 4 Department of Neuroradiology and Intracranial Endovascular Therapy, Alfried Krupp Krankenhaus, Essen, Germany

**Keywords:** Stroke, Thrombectomy, Single stentriever rescue, Endovascular treatment, Hemorrhage

## Abstract

**Background:**

Mechanical thrombectomy is the standard of care for acute ischemic stroke due to large-vessel occlusion; however, mechanical thrombectomy fails to achieve adequate recanalization in nearly one third of these cases. Rescue therapy using two stentrievers simultaneously yields good results in clots refractory to single stentriever treatment. We aimed to determine the safety and efficacy of first-line double stentriever thrombectomy for acute occlusion of the M1 segment of the middle cerebral artery and/or terminal internal carotid artery (TICA).

**Methods:**

This single-center study prospectively enrolled consecutive patients with a single M1/TICA occlusion to undergo double stentriever thrombectomy between May and October 2020. Outcomes included successful recanalization (modified thrombolysis in cerebral infarction, TICI 2b/3), first-pass effect, procedure times, number of device passes, symptomatic intracerebral hemorrhage, National Institutes of Health Stroke Scale Score (NIHSS) at discharge, 90-day functional independence (modified Rankin scale 0–2), and 90-day mortality.

**Results:**

We analyzed 39 patients median age 79 years (range 42–96 years); 23 (58.9%) female; 19 (48.7%) with TICA occlusions; 5 (12.8%) with mRS 3–5 at admission; mean NIHSS at admission, 17 ± 4.39). Mean time from symptom onset to final angiogram was 238.0 ± 94.6 min; mean intervention duration was 36.0 ± 24.2 min. The mean number of device passes was 1.5 ± 1.07. All patients had final TICI 2b/3, and 27 (69%) had TICI 2c/3 after the first pass. We observed 3 (7.9%) cases of intracerebral symptomatic hemorrhages. At 90 days, 16 (41%) patients were functionally independent and 9 (23%) had died. The percentage of patients with good clinical outcome at 90 days was 55.5% in the first-pass subgroup.

**Conclusion:**

Our findings suggest that first-line double stentriever thrombectomy is safe and effective for M1/TICA occlusions.

## Introduction

Recent trials have established the benefit of mechanical thrombectomy over medicinal management alone in patients with acute ischemic stroke from large-vessel occlusion of the anterior circulation, [1–7] where treatment aims to restore reperfusion by restoring blood flow through recanalization [8]; however, mechanical thrombectomy fails to achieve adequate recanalization in nearly one third of these patients [4], and the rates of effective recanalization remain low after rescue treatment, such as intra-arterial recombinant tissue plasminogen activator (rtPA), aspiration thrombectomy, mechanical thrombus disruption, and balloon angioplasty with or without stent placement [9–12].

Rescue therapy using two simultaneous stentrievers yields good results in patients with clots refractory to mechanical thrombectomy with a single stentriever [13–19]; however, the feasibility of this technique as a first-line treatment remains to be determined.

## Objective

This prospective study aimed to assess the efficacy and safety of first-line double stentriever thrombectomy for acute occlusion of the middle cerebral and/or terminal internal carotid arteries.

## Methods

### Study Design and Patient Selection

This prospective study analyzed clinical, radiologic, and safety outcomes in consecutive patients treated with mechanical thrombectomy using two stentrievers simultaneously as the first-line treatment for occlusions of the terminal internal carotid artery (TICA) and/or M1 segment of the middle cerebral artery at a single stroke center between May 2020 and October 2020. Patients with proximal tandem and posterior circulation occlusions were excluded. The institutional ethics board approved the study, and all patients or their legally authorized representative provided written informed consent.

### Baseline Characteristics

We analyzed the following baseline variables: age, sex, anticoagulant treatment, cardiovascular risk factors, pre-stroke modified Rankin Scale (mRS) score, initial National Institutes of Health Stroke Scale (NIHSS) score, and Alberta Stroke Program Early Computed Tomography Score (ASPECTS), the presence of CT perfusion mismatch, and treatment with intravenous recombinant tissue plasminogen activator (rtPA) before mechanical thrombectomy.

### Endovascular Procedure

**Fig. 1:**
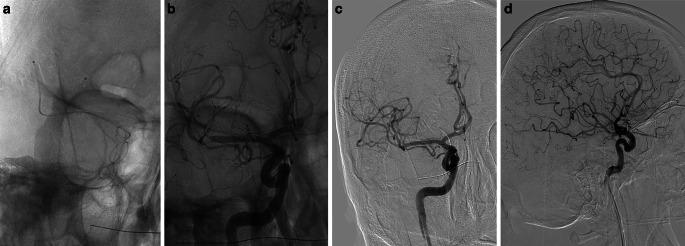
Occlusion of the M1 bifurcation treated with the dual stentriever technique. **a** Two microcatheters in the upper and lower trunks of the M2. **b** Injection of contrast material after the deployment of the two stentrievers. **c**, **d** Anteroposterior and lateral views of the final angiogram showing TICI 3 recanalization after the first pass

All procedures were performed using a biplane angiography system. An 8F balloon catheter was positioned in the internal carotid artery, and a 0.014-inch guidewire (Traxcess™, Microvention, Aliso Viejo, CA, USA) was advanced through a microcatheter (Phenom 21™, Medtronic, Irvine, CA, USA) to the target vessel. After passage through the clot, intra-arterial contrast medium was injected to verify the position of the microcatheter distal to the clot. Then, a second microcatheter was placed distally in the same artery or in another branch of the main artery. The stentrievers (Solitaire X 4 × 40 or 6 × 40 mm, Medtronic; Trevo NXT 4 × 35 or 6 × 30 mm, Stryker, Kalamazoo, MI, USA; or Embotrap II 5 × 37 or 6.5 × 45 mm, Cerenovus; Irvine, CA, USA) were deployed by withdrawing the microcatheters in a parallel or Y configuration and contrast material was injected to evaluate flow after placement of the devices. The proximal balloon guiding catheter was inflated 1–2min later to arrest blood flow, and the opened stentrievers were removed simultaneously with aspiration using a 60-mL syringe (Fig. 1). No aspiration catheters, combined techniques or adjunctive treatments were used.

### Procedural and Clinical Outcomes

Recanalization was measured with the thrombolysis in cerebral infarction (TICI) score, adjudicated by two independent physicians with extensive experience not included in the routine interventions of the patients.

Successful recanalization was defined as TICI score 2b/3 (complete or near-complete recanalization) and the first pass effect was defined as achieving a TICI 2c/3 after a single pass. We also analyzed the mean treatment time (stroke symptom onset to final angiogram), mean intervention time (arterial puncture to final angiogram), mean number of device passes, vasospasm after retraction of the device (defined as > 50% stenosis on follow-up angiograms), vessel perforation, contrast extravasation, and embolism in a new vascular territory.

Clinical and safety outcomes included symptomatic intracerebral hemorrhage (sICH, defined as any type of hemorrhage with an increase of ≥ 4 in NIHSS score), mean NIHSS score at discharge and functional independence (mRS score 0–2) and mortality at 90 days.

### Statistical Analysis

Descriptive statistics were generated using SPSS 17.0 software (IBM, Armonk, NY, USA). Categorical variables are presented as absolute values and percentages, and continuous variables are presented as means ± standard deviations or medians (ranges), as appropriate.

## Results

### Baseline Characteristics

We analyzed 39 patients (median age 79 years, range 42–96 years; 23, 58.9% women) who presented with a single TICA (*n* = 19; 48.7%) or M1 (*n* = 20; 51.3%) occlusion on initial angiograms. The most common cardiovascular risk factor was hypertension (*n* = 26; 66.6%), followed by atrial fibrillation (*n* = 12; 30.7%). Pre-stroke mRS scores indicated moderate to severe disability (mRS score 3–5) in 5 (12.8%) patients. At admission, mean NIHSS score was 17 ± 4.39, and median ASPECTS was 7 (range 4–10). A total of 13 (33.3%) were on anticoagulant or antiplatelet treatment, and 6 (15.3%) received at least 1 bolus of intravenous rtPA before the procedure. Table 1 provides details about patients’ baseline characteristics.

**Table 1:** Baseline patient characteristics

Characteristic	Overall (*N* = 39)
*Age*, in years	79 (42–96)
*Female*	23 (58.9%)
*Cardiovascular comorbidities*	
Hypertension	26 (66.6%)
Atrial fibrillation	12 (30.7%)
*mRS*	
0–2	34 (87.1%)
3–5	5 (12.8%)
*NIHSS*	
0	0 (0%)
1–4	0 (0%)
5–15	13 (33.3%)
16–21	26 (66.6%)
Mean ± standard deviation	17 ± 4.39
*ASPECTS*	7 (4–10)
*IV rtPA*	6 (15.3%)

Data are *N* (%), mean ± SD, or median (minimum–maximum)

*ASPECTS* Alberta Stroke Program Early CT Score, *IV rtPA* intravenous recombinant tissue plasminogen activator, *mRS* modified Rankin Scale, *NIHSS* National Institutes of Health Stroke Scale.

### Procedural Characteristics and Efficacy Outcomes

All patients were treated under conscious sedation. The mean time from symptom onset to final angiogram was 238 ± 94.6 min, with a mean intervention time of 36 ± 24.2 min. The mean number of device passes was 1.5. Target artery recanalization was assessed in all 39 patients, recanalization was successful (TICI 2b/3) in all patients and the first pass effect was achieved in 27 (69%) patients.

### Safety and Clinical Outcomes

Three hemorrhagic complications (7.9%) were symptomatic. No vasospasms were observed. Contrast extravasation without a visible arterial perforation was observed in 2 (5.8%) patients: 1 subarachnoid hemorrhage (Fisher grade 3) and 1 direct carotid-cavernous fistula. No embolisms in a new vascular territory were observed, but embolisms to the same territory were observed in 3 (7.6%) patients. The mean NIHSS score at discharge was 5.9 ± 7.17, 90 days after the procedure 9 (23%) patients had died, and 16 (41%) were functionally independent (mRS 0–2). The percentage of patients with good clinical outcome at 90 days was 55.5% in the first pass effect subgroup. Table 2 provides details about procedural and clinical outcomes.

**Table 2:** Procedural and clinical characteristics

Characteristic	Overall (*N* = 39)
*Treatment time*^a^* (min)*	238.0 ± 94.6
*Intervention time*^b^* (min)*	36.0 ± 24.2
*Device passes*	1.5 ± 1.07
1	27 (69.2%)
2	7 (17.94%)
3	2 (5.12%)
*Final TICI 2b/3*	39 (100%)
*Final TICI 2c/3*	34 (87%)
*Final TICI 3*	32 (82%)
*First-pass TICI 2b/3*	27 (69%)
*First-pass TICI 2c/3*	27 (69%)
*First-pass TICI 3*	25 (64%)
*Symptomatic intracerebral hemorrhage*	3 (7.6%)
*Procedural complications*	
Contrast extravasation	2 (5.8%)
Embolism in new vascular territory	0 (0.0%)
*NIHSS at discharge (N* *=* *35)*	5.9 ± 6.17
0	7 (20%)
1–4	12 (34.2%)
5–15	13 (37.1%)
16–21	3 (8.5%)
*90-day mortality*	9 (23%)
*90-day mRS 0–2*	16 (41%)
*90-day mRS 0–2 ***(**first-pass TICI 2b/3 subgroup)	15/27 (55.5%)

Data are *N* (%) or mean ± SD

*TICI* thrombolysis in cerebral infarction score, *NIHSS* National Institutes of Health Stroke Scale, *mRS* modified Rankin Scale

^a^ Treatment time is defined as the time from stroke symptom onset to final angiogram

^b^ Intervention time is defined as the time from arterial puncture to final angiogram

## Discussion

The double stentriever thrombectomy has been initially found out by T. Liebig (Department of Neuroradiology, TUM, Munich, Germany) and its primary systematic use in the treatment of large vessel occlusion by R. Chapot (Alfried Krupp Krankenhaus, Essen, Germany). The high rates of recanalization, especially after the first pass, the low rate of complications, and 90-day mortality rate in line with those reported in previous clinical trials with other mechanical thrombectomy techniques indicate that this promising new approach merits further investigation. First-line double stentriever thrombectomy for TICA and M1 occlusions achieved successful recanalization (TICI 2b/3) in all patients, representing a notably higher rate of successful recanalization than reported with standard stentriever techniques alone (58%–83%) [1, 4, 20], or with the direct aspiration first-pass thrombectomy technique with large-bore aspiration catheters alone (79%–83%) [20, 21]. In another study involving a retrospective analysis of 200 patients treated with stent retriever-assisted vacuum-locked extraction (SAVE) technique due to intracranial large vessel occlusion, Maus et al. documented 57% of first pass effect (TICI 2c/3) [22]. The efficiency of double stentriever thrombectomy seems however to be even higher with a 69% first pass effect rate to achieve TICI 2c/3. This rate is higher than the rates for standard stentriever techniques reported by Zaidat et al. [23] (64% and 10% in M1 and TICA occlusions, respectively), by MR CLEAN Investigators [24] (24%) and in the meta-analysis published by Bai et al. [25] (49.7%). The rate of patients with mRS 0–2 at 90 days in our series was 41%, similar to what was reported in the NASA Registry [24]. The percentage of patients with good clinical outcome was 55.5% in the first-pass effect subgroup, similar to the results reported by the Den Hartog et al. [25] (56%), higher than what was observed in the meta-analysis of Bai et al. [25] and lower than what was described by Zaidat et al. (61.3%) [23]; however, the rate of patients with mRS 0–2 at days was not better compared to the results from some randomized trials (e.g., ESCAPE 53%, EXTEND-IA 71%) [3, 26]. We have to take into account that pre-stroke mRS scores indicate moderate to severe disability (mRS score 3–5) in 5 (12.8%) of our patients.

Various authors have analyzed double stentriever thrombectomy as a rescue therapy in bifurcation occlusion [13–19]. With the exception of Li et al. [18], all these reports were retrospective and included few patients. The rates of successful recanalization were high (80%–85% [14, 15, 18, 19]), but lower than ours. The only study that analyzed recanalization after the first pass, Cabral et al. [19], reported 75% success, higher than the 69% in our series. Those authors used the combined stentriever technique as rescue and not as primary technique. None of these studies reported serious complications from using two devices simultaneously. It reinforces the advantage of using this technique as a first line treatment.

Advantages of using a second device include better integration of the thrombus thanks to increased radial force from the second stentriever and more complete coverage of the thrombus [15]. Integration of the thrombus increases the retrieval force transmitted to the thrombus and improves the likelihood of successful thrombectomy. The dual stentriever technique may be particularly helpful for clots involving arterial bifurcations, which are often resistant to multiple passes of a single stentriever [3, 17], and the increased radial force from a second stentriever might also be especially beneficial in hard, fibrin-rich thrombi that are difficult to penetrate with a single device [15].

Possible disadvantages of the double stentriever technique include trauma to intracranial vessels, a potentially serious adverse event in mechanical thrombectomy that is uncommon in procedures performed with a single stentriever [27, 28]. The reported incidences of arterial dissection, symptomatic cerebral hemorrhage and subarachnoid hemorrhage were 4.5%, 1.1% and 1.1%, respectively. Using two stentrievers may potentially increase the force on the endothelium during the retrieval maneuver [14]. In our study, we observed sICH in 7.6% of patients and subarachnoid hemorrhage in 5.1%, these rates are lower than in reports where the technique was used in rescue therapy (10% sICH reported Klisch et al. [14] and Cabral et al. [19] and 10.7% subarachnoid hemorrhage in Li et al. [18]). We also observed extravasation of contrast material in two patients. One of these resulted in a Fisher grade 3 subarachnoid hemorrhage and the other in a direct carotid-cavernous fistula. Extravasation of contrast to the subarachnoid space is a complication we can observe occasionally even after the use of one stentriever and is believed to be due to the traction of the arteries during the extraction of the device [27]. Direct fistula is an unusual complication that happened in a patient in his 90s with a severely calcified carotid siphon. One possible explanation is that a stentriever was caught in a calcified plaque that was torn causing the fistula. Both resolved spontaneously after the proximal balloon was inflated for a few minutes without worsening the clinical situation of the patients. The limited literature of the double stentriever technique [15, 18, 19] suggests it is well-tolerated with a risk profile similar to conventional single stentriever technique. Klisch et al. [14] described 1 case of vessel perforation and 7 cases of mild vasospasm during mechanical thrombectomy using the double stentriever technique as a rescue therapy in 10 patients. Other disadvantages are the added complexity of using two stentrievers, which requires a higher level of operator experience, and potential increase in costs from using two stentrievers.

The present study has several limitations. First, the sample was relatively small. Given the scant corroborating evidence for the safety and efficacy of our treatment protocol, additional data is necessary before double stentriever thrombectomy can be included as a first-line therapeutic option for proximal occlusion in international guidelines. Second, patients in this prospective case series were not randomized to receive first-line double stentriever thrombectomy, and there was no control group for comparison. It is essential to carefully assess the possible risks and benefits of this approach before attempting it in individual patients. Randomized controlled trials comparing outcomes after mechanical thrombectomy using two stentrievers versus using a single stentriever will further clarify the effectiveness, safety and cost of this promising technique.

## Conclusion

We found that double stentriever thrombectomy yielded high rates of successful recanalization after the first pass with a low rate of complications, suggesting it can be an effective and safe first-line treatment for M1 and TICA occlusions. Nevertheless, larger prospective studies are required to validate the feasibility and safety of this strategy and further determine its ability to improve clinical outcomes.

## Abbreviations

ASPECTS: Alberta Stroke Program Early Computed Tomography Score
NIHSS: National Institutes of Health Stroke Scale
rtPA: Recombinant tissue plasminogen activator
TICA: Terminal internal carotid artery
